# Ultrasound-guided versus low dose computed tomography scanning guidance for lumbar facet joint injections: same accuracy and efficiency

**DOI:** 10.1186/s12871-018-0620-7

**Published:** 2018-11-07

**Authors:** Ling Ye, Chuanbing Wen, Hui Liu

**Affiliations:** 1 Department of Pain management, West China Hospital, Sichuan University, Chengdu, Sichuan Province 610041 People’s Republic of China; 2 Department of Pain Management, Sichuan Academy of Medical Sciences & Sichuan Provincial People’s Hospital, Chengdu, Sichuan Province 610072 People’s Republic of China

**Keywords:** Ultrasound, Computed tomography, Accuracy, Efficiency, Lumbar facet joint injection

## Abstract

**Background:**

The purpose of this study was to investigate the feasibility, accuracy and efficiency of the facet joint injections in the lumbar spine by ultrasound guided versus lose dose computed tomography (CT) guidance.

**Methods:**

First the examination on the joint space of the facet joints of the lumbar spine was obtained by the ultrasound in 10 patients. Second forty patients were randomized assigned into two groups: ultrasound group and low dose CT group. Comparison was made in the clinical efficiency between the ultrasound-guided group and CT group. The feasibility, accuracy and efficiency of the ultrasound-guided lumbar facet joint injections were also evaluated.

**Results:**

A total of 88 lumbar facet joints from L_1_ to S_1_ were clearly visualized in the 10 patients. Both the ultrasound and the CT measurements showed the same average depth and lateral distance to the reference point (*P* > 0.05). And 86.5% of the facet joint injections (64/74) were correctly performed under the ultrasound guidance in the first time. The exact placement of the needle tips was evaluated by CT. After the lumbar facet joint injections, the clinical efficiency was almost the same in the ultrasound-guided group as in the CT group.

**Conclusions:**

The lumbar facet joint space can be accurately demonstrated by ultrasound. The ultrasound-guided facet joint injection in the lumbar spine obtained almost the same satisfactory feasibility, accuracy and clinical efficiency compared with low dose CT. Ultrasound technique could provide the real-time monitoring.

**Trial registration:**

This study was registered on Chinese Clinical Trial Registry (ChiCTR1800018819, retrospective registered on 11/10/2018).

## Background

The facet joint-related pain is very common, and it has been identified as a common source of the low back pain [1–3]. However, we cannot diagnose it solely based on physical examination [4] or radiographic imaging [5]. Facet joint block is a commonly used method for relieve the low back pain, and for diagnosis and treatment of the facet joint-related pain [6]. Facet joint blocks are usually performed with the help of the fluoroscopic guidance or the computed tomography (CT) scanning guidance for a precise localization of the needle tips and avoidance of complications [7–9]. But the two techniques are inevitably associated with significant radiation doses for both the patient and the pain physicians [10].

Ultrasound is not associated with an exposure to radiation, and equipment is not too expensive, which is portable and can be used as a real-time monitoring image guide tool. Ultrasonography has been applied for guidance in nerve blocks [11–15]. Ultrasound can exactly indicate the injection sites and monitor the needle insertion and the spread of local anesthetics in real time. The success and validity of the lumbar facet joint injections may depend on the accurate insertion of the needle tip. Inaccurate positioning of a needle tip may result in an inadvertent spread of the local anesthetic into the intervertebral foramen, the epidural space, or even the subarachnoid space, which can cause some serious complications [16, 17].

The present study was designed to evaluate the feasibility and accuracy of the ultrasound-guided lumbar facet joint injections, which were compared with CT. The pain relief was also assessed.

## Materials and methods

The study was approved by the institutional ethics committee of West China Hospital, Sichuan University (Chengdu, China). Informed consents were obtained from all the participants. All the procedures were performed by the same group of doctors.

### The first part - the CT analysis study

Ten adult patients with low back pain who were required CT scan (5 women, 5 men) were enrolled between Jan 3, 2016 to March 3, 2016 in West China Hospital, Sichuan University, Chengdu. All the patients met the following inclusion criteria: 18–80 years old. The patients were excluded as following: the body mass index (BMI) ≥ 25 kg/m^2^; spinal deformities including congenital scoliosis and kyphosis, secondary deformities such as ankylosing spondylitis, spinal tuberculosis, neuromuscular scoliosis, Scheumann disease and osteoporosis.

**Fig. 1:**
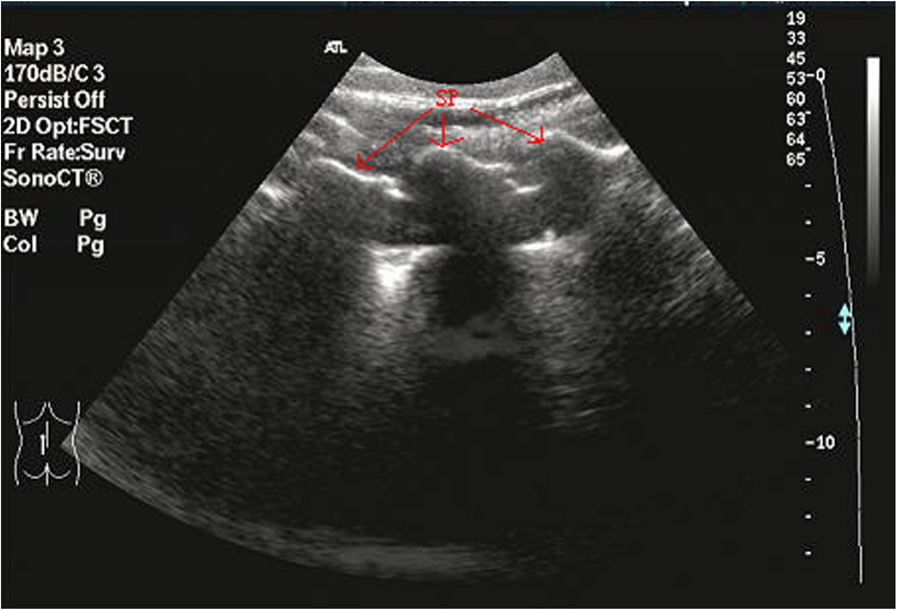
The spinous processes of the lumbar spine demonstrated in a posterior paravertebral parasagittal sonogram. Arrow: spinous process (SP)

All the demographic data were recorded. The patients were placed in a prone position, with the abdomen supported by the pillows to compensate for the lumbar lordosis. Ultrasound examinations were performed by one ultrasound investigator experienced in the musculoskeletal ultrasound examination, and a standard ultrasound device (Philips, HDI 3500 or 5000) was used, which used a broadband curved array transducer working at 3–5 MHz and a broadband linear array working at 12–15 MHz. To identify the spinal levels, the posterior parasagittal sonograms were obtained at levels L1 to S1 (Fig. 1) [18–20].

**Fig. 2:**
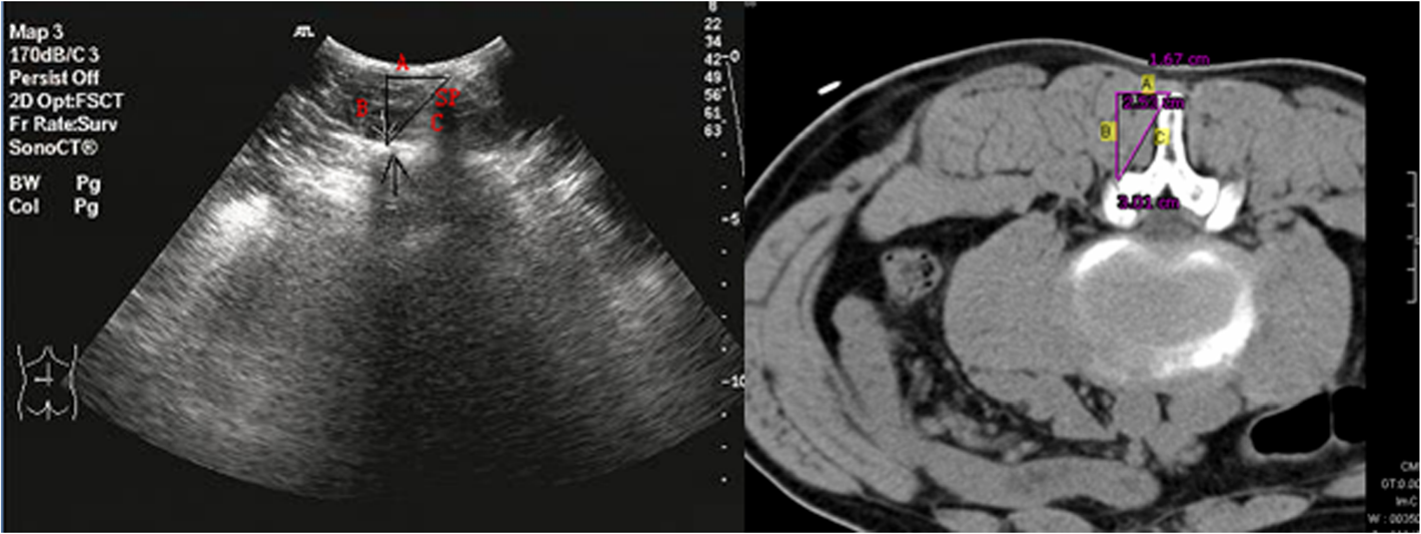
The lateral distance (**a**) defined as the horizontal distance from the middle point between the tips of the spinous processes to the reference point, the depth (**b**) defined as the vertical distance from the middle of the tip of the spinous process to the reference point, and the oblique line (**c**) defined as the distance from the middle point between the tips of the spinous processes to the reference point

To get the ultrasound view, first the transducer was placed on the long axis of the facet column, which appears like a camel’s hump. Then the transducer was rotated 90°to get the short axis view. The lumbar facet joints were delineated with the help of the transverse sonograms at each level. The transducer was first placed in the midline for scanning the short axis view of the lumbar spine. Then the transducer is relocated more cranially until spinous process was seen in the middle of the view. The lateral border comprises bilateral inferior articular process, superior articular process and transverse process. The sonogram of each plane was measured by the ultrasound measuring device. The lateral distance (A) was defined as the horizontal distance from the middle of the tip of the spinous process to the reference point; the depth (B) was defined as the vertical distance from the middle point between the tips of the spinous processes to the reference point; the oblique line (C) indicates the distance from the middle point between the tips of the spinous processes to the reference point. The above three distances were measured to assess the position of the facet joint space in the transverse sonograms. The distances A, B and C of each sonogram were evaluated by a spiral CT (low dose, 100 kV, 35 mAs) on the same plane with the same approach, reformatted to 1-mm axial slices (Fig. 2). All the values are presented as means ± SD.

### The second part – The clinical study

**Fig. 3:**
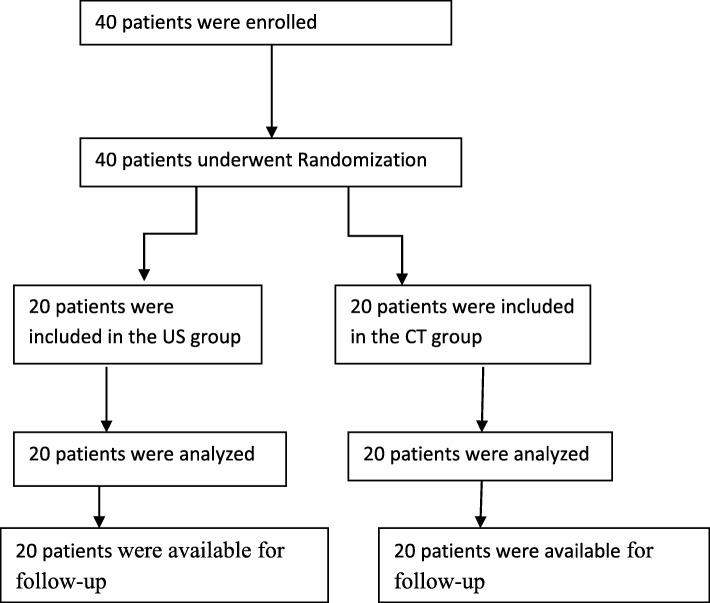
Patient flow chart: randomization, treatment, and inclusion in analysis

Forty adult patients (20 women, 20 men) were consecutively enrolled between April 1, 2016 to Dec20, 2016 in West China Hospital, Sichuan University, Chengdu (Fig. 3). The inclusion criteria were as following: 18–80 years old; having undergone CT or MRI of their lumbar spine, with the visible lumbar facet joint spaces. The patients were excluded as following: any potential contraindications, such as a spinal tumor, spinal deformities, spinal instability, discitis, and fracture; local or systemic infection or spinal infections; allergy to steroids or anesthetics; previous surgery; uncorrectable coagulopathy; pregnant; BMI ≥ 25 kg/m2. Based on the computer-generated randomization table, the patients were randomized assigned to two groups: patients in the group 1 were scheduled for the ultrasound-guided infiltrations (the US group), patients in the group 2 were scheduled for the lose dose CT guidance (the CT group, low dose, 100 kV, 35 mAs).

### The US group

The patients were placed in a prone position with the abdomen supported by the pillows. One doctor experienced in the musculoskeletal ultrasound performed the ultrasound-guided facet joints injection in the lumbar spine in the US group (20 patients). This ultrasound-guided approach to the facet joint blocks was as the same as that in the first part. The skin was routinely sterilized. A spinal needle (20 gauge, 90 mm) was inserted into the ideal target position. When the needle tip was properly placed, 2 ml of a mixture that contained 0.5 ml of 2% lidocaine, 0.4 mg of compound betamethasone was injected into the facet joint space.

### The CT group

**Fig. 4:**
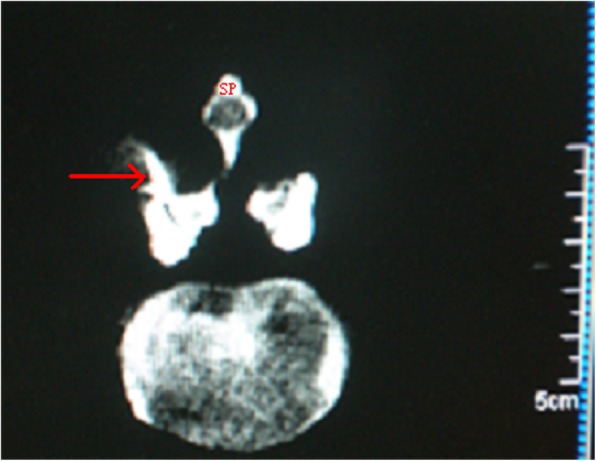
The needle tip verified under the CT monitoring. SP: Spinous process; arrow: needle

The patients were placed in the same position as in the US group. The facet joint space was identified as did in the first part study described above. The needle tip was verified under the CT monitoring (Fig. 4) [18–20]. Then, the same drug was injected in the facet joint space as that in the US group.

### Measurements

In both the groups, the visual analog scale (VAS) score regarding the low back pain before the facet joint injections were recorded. VAS and the remission rate (VAS < 3) of half an hour, one day, two days, 6 weeks after the procedures were recorded. The accuracy rate of the ultrasound procedure was defined as the percentage of the facet joint space surveyed by ultrasound. The levels of the facet joint injections were recorded in the two groups. The achievement rate in the US group was also recorded.

### Statistical analysis

On the basis of our pilot study data, A sample size of 18 allowed the detection of a 20% difference in the proportion with an α 0.05 (two-tailed) and a β of 0.20, power of 0.8. To account for attrition, a sample size of 20 was selected for each group.

Statistical analysis was performed with SAS and spss17.0. The features of the patients in the two parts were presented as medians (ranges). The distances are expressed as means ± SD (ranges), and they were analyzed for normality by means of the multivariant matched-pairs t test the A, B and C values in the volunteer study. *P* values less than 0.05 were considered statistically significant.

## Results

### The first part – The CT analysis study

In all the patients (5 women, 5 men; median age, 56.2 ± 14.8 kg; height, 159.1 ± 6.56 cm; weight, 56.6 ± 4.11 kg; BMI, 22.37 ± 1.21), there were 88 facet joints that were entirely visible, accounting for 88%. Ultrasound and CT showed the same mean values of distances A, B and C(*P* > 0.05) (Table 1). In three patients, 12 facet joints could not be identified for providing a lumbar approach to the facet joint by ultrasound, but they could be identified by CT.

**Table 1:** A, B, C values of ultrasound and CT in the same spine level

Ultrasound			CT	
	Left(cm)	right(cm)	left(cm)	right(cm)
AL1/2	1.56 ± 0.19	1.54 ± 0.25	1.55 ± 0.21	1.51 ± 0.23
BL1/2	2.17 ± 0.22	2.16 ± 0.28	2.17 ± 0.21	2.17 ± 0.32
CL1/2	2.67 ± 0.26	2.66 ± 0.26	2.68 ± 0.25	2.65 ± 0.31
AL2/3	1.54 ± 0.22	1.58 ± 0.28	1.51 ± 0.21	1.56 ± 0.26
BL2/3	2.35 ± 0.25	2.39 ± 0.24	2.39 ± 0.26	2.41 ± 0.23
CL2/3	2.84 ± 0.26	2.87 ± 0.26	2.85 ± 0.24	2.88 ± 0.24
AL3/4	1.77 ± 0.32	1.79 ± 0.28	1.76 ± 0.32	1.82 ± 0.30
BL3/4	2.46 ± 0.23	2.46 ± 0.24	2.49 ± 0.23	2.47 ± 0.22
CL3/4	3.05 ± 0.23	3.05 ± 0.23	3.06 ± 0.23	3.08 ± 0.22
AL4/5	1.97 ± 0.32	2.03 ± 0.32	1.97 ± 0.32	2.03 ± 0.32
BL4/5	2.27 ± 0.37	2.29 ± 0.35	2.28 ± 0.42	2.30 ± 0.33
CL4/5	3.06 ± 0.23	3.07 ± 0.33	3.08 ± 0.24	3.08 ± 0.31
AL5S1	2.12 ± 0.35	2.15 ± 0.29	2.11 ± 0.37	2.16 ± 0.31
BL5S1	2.21 ± 0.33	2.25 ± 0.23	2.22 ± 0.35	2.26 ± 0.27
CL5S1	3.08 ± 0.26	3.09 ± 0.31	3.09 ± 0.26	3.15 ± 0.34

### The second part – The clinical study

All the patients in the two groups suffered from chronic low back pain, with visual analogue scale (VAS) > 3(0–10) (the US group:7.00 ± 0.88; the CT group: 6.25 ± 2.31; *P* > 0.05) before the blocking procedure. There was no significant difference between the two groups (Table 2). An obvious paravertebral lumbar tenderness was found by ultrasound in the 20 patients, involving 74 facet joints associated with the injection, which were confirmed by the CT scan. 86.5% of the needles (64/74) could be successfully guided by ultrasound into the right facet joint space in the first time. In some patients, adaptation was necessary for the needling during the ultrasound guidance. Under CT, only 10 of the 74 needles had to be slightly corrected in position. No signs of the nerve root block, and no other neurological symptoms were observed. Half an hour after the injections, 14 patients had a reduction in the pain, with a remission rate ≥ 50% in the US group, and 12 patients, in the CT group.

**Table 2:** Demographic data of the two groups

	US group	CT group	P
F/M	9/11	12/8	
Years	55 ± 12.4	54.5 ± 14.4	*P* > 0.05
Disease course (mon)	52.6 ± 11.2	42.6 ± 5.2	*P* > 0.05
BMI	24.3 ± 0.80	24.7 ± 2.19	*P* > 0.05
VAS	7.00 ± 0.88	6.25 ± 2.31	*P* > 0.05

*F* female, *M* male, *BMI* body mass index, *VAS* visual analog scale

In the US group, 30 min, 1 day, 2 days and 6 weeks after the procedures, there were 16, 18, 18 and 18 patients who had a decrease (≥3) in the VAS score respectively, and there were 14, 16, 16 and 16 patients had a reduction in the pain severity respectively, with a remission rate ≥ 50%, respectively; the follow-up after 6 weeks revealed a 73% pain remission rate (Table 2). Only 2 patients reported pain aggravation 30 min after procedure and then relived after 1 day.

In the CT group, 30 min, 1 day, 2 days and 6 weeks after the puncture procedures, there were 15, 16, 16 and 16 patients who had a decrease (≥3) in the VAS score respectively, and 14, 16, 16 and 16 patients who had a reduction in the pain severity respectively, with a remission rate ≥ 50%, respectively; the follow-up after 6 weeks revealed a 57% pain remission rate (Table 3). There were 4 patients reported pain aggravation 30 min after procedure and then relived after 1 day.

**Table 3:** Patients of remission rate ≥ 50% and VAS after procedures

	Remission rate		VAS	
	US group	CT group	US group	CT group
30 min	14	14	2.95 ± 0.18	2.98 ± 0.21
1 day	16	16	2.76 ± 0.14	2.98 ± 0.18
2 days	16	16	2.81 ± 0.20	2.83 ± 0.17
6 weeks	16	16	2.86 ± 0.15	2.84 ± 0.15

## Discussion

In the present study, the lumbar facet joint space can be accurately demonstrated by the ultrasound. The feasibility, accuracy and clinical efficiency of the ultrasound-guided approach for the lumbar facet joint injections were very satisfactory for the patients with low back pain.

The facet joints are often affected by the mechanical derangements or the degenerative alterations; thus, the reflex muscular spasm or the referred pain can be easily developed [21]. The facet syndrome has been defined as a lumbosacral pain with or without a sciatic pain, particularly associated with a twisting or rotary strain of the lumbosacral region. The pain can be unilateral or bilateral and is typically enhanced by hyperextension of the lumbar spine or the locally applied pressure on the facet joints. No specific anatomic or radiologic findings have been confirmed to be correlated with the clinical diagnosis of the facet syndrome. Consequently, the primarily-diagnostic facet joint blocks are required in many patients, using a fluoroscopy device or a CT scan or in the blind manner based on the indication by the X-ray examination.

The advantages of the ultrasound guidance include (but not limited to) an increased success rate, decreased complications caused by the needle malpositioning, a faster effect of the blocks, and a reduced amount of the local anesthetics [21–27]. Besides, no exposure to radiation for the patient and the doctor is an important advantage, which makes the ultrasound guidance applicable for the pregnant patient. As we know, fluoroscopy has a complication rate of 5–10%, and CT has a complication rate about 0.5%. The previous researches revealed some life-threatening complications caused by the fluoroscopy-guided infiltrations, such as pleural perforation and pneumothorax [7]. The ultrasound guidance is useful in facilitating peripheral and neuraxial blocks and offers the direct visualization of the target, adjacent structures, and local anesthetic spread [7, 28].

Kullmer, et al. described the ultrasound use for the facet joint infiltration of the lumbar spine only for the periarticular region, but they could not ensure the precise application of the intraarticular local anesthetic without the fluoroscopy monitoring or the use of the contrast media [25]. The facet joint blocks are mainly used for diagnosis and the needle placement, and a small volume of the local anesthetic is necessary to minimize the rate of the false-positive block or complication. Theoretically, the facet joint infiltration in the periarticular region may cause a false-positive result because of the aberrant local anesthetic spread (epidural, nerve root, multifidus muscle). The direct intraarticular injection is considered indispensable for treatment of the facet joint-related pain. 27 The recent researches presented the new methodology of the ultrasound-guided lumbar facet nerve block and lumbar facet joint infiltration in the cadavers or the patients in the first part [29–31].

The present study showed that the ultrasound could identify the lumbar facet joint space exactly in the patients, with advantages of greater feasibility, accuracy and clinical efficiency for the lumbar facet joint block.

The result indicated that this new method can provide a clear delineation of the target lumbar facet joint space and can guide the needle into the space. The placement of the needle can also be monitored by ultrasound in real time from the skin puncture to the final target space. The clinical efficiency was greater in the ultrasound-guided group than in the bland-manner group after the lumbar facet joint injections.

Confirmed by CT, 88 of the 100 lumbar facet joints of our patients could be precisely identified and visualized by ultrasound, and only 12 facet joints could not be identified. Based on the review by CT, the reason for the failure to identify the facet joints was that serious hyperosteogeny existed, which could not allow the facet joints to be visible. So, the patient with serious hyperosteogeny is unsuitable for this new method.

In the study, 32 of the 37 needle placements were correct in the first time, and the ultrasound-guided facet joint injections could be well performed. When the target structures were visualized, the needle could be advanced to the target structures exactly and safely.

Compared with fluoroscopy or CT scanning, ultrasound is not so expensive for use by the patient, and it can offer more flexibility in the clinical application. Meanwhile, ultrasound as a standard technique can provide the same accuracy for the needle placement. However, these advantages should be further confirmed by a still larger size of the samples.

Ultrasound can be applied for the steroid injections and for the diagnostic blocks. We still require sufficient data about the optimal volume for the facet joint injections. As we know, the usual capacity of the facet joint is 1–2 ml, so the injection of more than 2 ml may lead to an extracapsular leakage of the local anesthetic. The steroids given into the epidural space or neuroforamen may have some therapeutic effects. So, the 2-ml injection volume should be used. A significant difference in the pain relief was found between the two groups immediately after injection and during the follow-up (*P* < 0.05). Ultrasound, a safe and accurate guiding tool, has been used in our present clinical practice.

Meanwhile different kinds of ultrasound guided methods for relief of the facet joint pain has different advantages. In recent years, ultrasound has been widely used in viewing axial spines to get more comprehensive view of the spinous process, facet joint and transverse process [32, 33]. Chang KV et al. propose another ultrasound-guided approach which ultrasound guided the needle to the desired area on the long aixs and the confirmed the needle tip short axis [34]. The new method may be more suitable for L4/5 and L5/S1facets. In our study we get the view on the long axis of the facet column, then the transducer was rotated 90°to get the short axis view. And we tried to calculate the angle of spinous process to facet joint. So the view on short axis might be more comprehensive.

There were some limitations in the study. First, a limitation of our design was to include patients from 18 to 80 years and there maybe some differences in the anatomy, pain pattern and response to treatment in this wide ranging group. Another trial would be carried out in which we would try to divide the patients into two groups according to the age: group 1 including patients from 18 to 40 years; group 2 including patients from 41 to 80 years. Second, 2 patients (5 facet joints) needed the replacement of the needles even though the facet joints could precisely be identified and visualized. The reason was that the two patients had a very high muscular tone because of their nervousness. The needle could not be in line with the guided dotted line through the facet joint space on the screen, and we could not easily control the needle placement. Third, two patients had the increased pain scores 30 min after the injections in the blind-manner group. The reason was traced to the fact that the needle placement was difficult to be performed by the doctor, who performed puncture procedure repeatedly and the periarticular region was injured, which resulted in the increased degree of the pain. So, the ultrasound-guided facet joint injection can decrease the iatrogenic injury to the patient.

## Conclusion

In conclusion, the lumbar facet joint space can be accurately demonstrated by the ultrasound which was confirmed with CT scan. The real-time ultrasound guidance for the needle can be performed. The feasibility, accuracy and clinical efficiency of the ultrasound-guided approach for the lumbar facet joint injections are very satisfactory for the patients with a low back pain.
